# Structural MRI Reveals Cervical Spinal Cord Atrophy in the P301L Mouse Model of Tauopathy: Gender and Transgene-Dosing Effects

**DOI:** 10.3389/fnagi.2022.825996

**Published:** 2022-05-02

**Authors:** Thomas Sartoretti, Robert P. Ganley, Ruiqing Ni, Patrick Freund, Hanns Ulrich Zeilhofer, Jan Klohs

**Affiliations:** ^1^Institute for Biomedical Engineering, ETH & University of Zurich, Zurich, Switzerland; ^2^Institute of Pharmacology and Toxicology, University of Zurich, Zurich, Switzerland; ^3^Zurich Neuroscience Center (ZNZ), Zurich, Switzerland; ^4^Balgrist University Hospital, University of Zurich, Zurich, Switzerland; ^5^Institute for Pharmaceutical Sciences, ETH Zurich, Zurich, Switzerland

**Keywords:** tau, spinal cord, magnetic resonance imaging, frontotemporal dementia, Alzheimer’s disease, animal models

## Abstract

In primary tauopathies, the deposition of tau neurofibrillary tangles and threads as well as neurodegenerative changes have been found within the brain and spinal cord. While degenerative changes have been intensively studied in the brain using structural magnetic resonance imaging (MRI), MRI studies investigating the spinal cord are still scarce. In the present study, we acquired *ex vivo* high resolution structural MRI of the cervical spinal cord of 8.5–9 month old hemizygous and homozygous P301L mice and non-transgenic littermates of both genders. We assessed the total cross-sectional area, and the gray and white matter anterior-posterior width and left-right width that are established imaging marker of spinal cord degeneration. We observed significant tissue-specific reductions in these parameters in female P301L mice that were stronger in homozygous than in hemizygous P301L mice, indicating both an effect of gender and transgene expression on cervical spinal cord atrophy. Moreover, atrophy was stronger in the gray matter than in the white matter. Immunohistochemical analysis revealed neurodegenerative and neuroinflammatory changes in the cervical spinal cord in both the gray and white matter of P301L mice. Collectively, our results provide evidence for cervical spinal cord atrophy that may directly contribute to the motor signs associated with tauopathy.

## Introduction

Primary tauopathies are characterized by the aggregation of insoluble, hyperphosphorylated tau protein into intraneuronal neurofibrillary tangles, threads, and inclusions in neurons and glia (Lee et al., 2001). This group includes progressive supranuclear palsy, corticobasal degeneration, and a number of sporadic and familial frontotemporal dementias and Alzheimer’s disease, that ketamine acts on postsynaptic neurons where beta-amyloid is also deposited into extracellular plaques in addition to tau (Grundke-Iqbal et al., 1986). In healthy neurons, the tau protein stabilizes axonal microtubules that are necessary for proper neuronal function, but in Alzheimer’s disease and tauopathies, there is an abnormal accumulation of tau protein in somatodendritic compartments, associated with axonal and synaptic dysfunction, neuronal loss, atrophy, and cognitive decline (Lee et al., 2001). Tauopathy spreads sequentially across the brain, through neuroanatomically and functionally connected areas (Braak and Braak, 1991; Vogel et al., 2020).

Tauopathy is also known to extend into the spinal cord (SC). Neuropathological studies have shown that the SC of patients with Alzheimer’s disease and variants of the disease have tau neurofibrillary tangles and threads in the SC (Schmidt et al., 2001; Rudzinski et al., 2008; Dugger et al., 2013). Neuropathological findings were most pronounced in the cervical SC, with a progressive decrease at the thoracic and lumbar level (Dugger et al., 2013). In addition, there is marked degeneration of the corticospinal tract with myelin damage and gliosis (Verkkoniemi et al., 2001; Rudzinski et al., 2008). Furthermore, Alzheimer’s disease and tauopathy patients have, in addition to cognitive impairment, characteristic motor signs, including motor slowing, rigidity, gait and posture abnormalities, bradykinesia and tremor, which are related to worsened clinical outcome (Scarmeas et al., 2005; Waite et al., 2005; Josephs, 2017). Related studies in animal models of tauopathy have shown the deposition of phosphorylated tau and neurofibrillary tangles in the SC in several different strains (Ishihara et al., 1999; Spittaels et al., 1999; Duff et al., 2000; Probst et al., 2000; Götz et al., 2001; Allen et al., 2002; Higuchi et al., 2002; Lin et al., 2003, 2005; Zehr et al., 2004; Yoshiyama et al., 2007). Moreover, these studies provided evidence for neuronal degeneration, myelin damage, and inflammation. The SC pathology was accompanied by motor signs such as paresis, tremor, and muscle weakness (Duff et al., 2000; Probst et al., 2000; Allen et al., 2002; Higuchi et al., 2002; Lin et al., 2005). Thus, the SC is a clinical eloquent site in the pathology and symptomatology of tauopathies.

High-resolution structural magnetic resonance imaging (MRI) is widely applied in clinical studies to assess degenerative changes of the brain (Klohs and Rudin, 2011). Such studies revealed distinct pattern of gray and white matter atrophy in the brain of patients with progressive supranuclear palsy (Price et al., 2004), corticobasal degeneration (Tokumaru et al., 2009), Pick’s disease (Lieberman et al., 1998), frontotemporal dementia (Ghetti et al., 2008; Rohrer et al., 2010), and Alzheimer’s disease (Holland et al., 2009; Shi et al., 2009; Tabatabaei-Jafari et al., 2019; Lorenzi et al., 2020). Importantly, regional atrophy is detectable in longitudinal studies within short time intervals (Holland et al., 2009; Rohrer et al., 2010), is a marker of cognitive decline (Tabatabaei-Jafari et al., 2019), and distinguish between different tauopathies and other causes of neurodegeneration (Price et al., 2004; Ghetti et al., 2008; Shi et al., 2009; Tokumaru et al., 2009; Rohrer et al., 2010). Related MRI studies in animal models of tauopathies have also revealed cerebral atrophy, for example atrophy was found in the hippocampus and cortex in the rTg4510 tau mouse model (Xie et al., 2010; Yue et al., 2011; Holmes et al., 2017; Ma et al., 2019).

Despite the involvement of the SC in tau pathology, structural MRI studies of the SC in patients and animal models are still scarce. A recent study has reported atrophy of the cervical SC in Alzheimer‘s disease patients (Lorenzi et al., 2020). The study reported reduced cross-sectional areas of C1 to C5 and reduced cross-sectional volumes in C1 and C2. However, the tau pathology has been identified as a major driver of local neurodegeneration in Alzheimer‘s disease (Iaccarino et al., 2018; Joie et al., 2020). This has prompted us to investigate morphological changes in the cervical SC in the P301L mouse model of four-repeat taupoathy (Götz et al., 2001). We acquired *ex vivo* high-resolution structural MRI data of the cervical SC of 8.5–9 months old P301L mice and non-transgenic littermates and assessed morphometric metrics, that are established imaging markers of SC degeneration (Mann et al., 2007; Ellingson et al., 2010; Seif et al., 2018). To test for transgene-dose-dependent effects, we assessed morphometric metrics for both hemizygous and homozygous P301L mice and compared them to non-transgenic littermates. We hypothesized that effects on morphometric parameters would be stronger in homozygotes than in hemizygotes, because of the stronger tau transgene expression in homozygous P301L mice. Moreover, given the reported effects of gender on tau pathology in animal models of tauopathy (Yue et al., 2011; Sun et al., 2020), we examined the effect of gender on the measured morphological parameters of the cervical SC.

## Materials and Methods

### Animal Model

P301L mice expressing the human four-repeat tau isoform under control of the murine Thy 1.2 promoter were backcrossed with C57BL/6J mice for >20 generations and maintained on a C57BL/6J background [B6.Dg-Tg(Thy1.2-TauP301L)183 Nitsch] (Götz et al., 2001). The strain expresses the human four-repeat tau isoform along with human pathogenic mutation P301L under the neuronal Thy 1.2 promoter. P301L mice show high expression of human P301L tau in neurons and show abnormal filaments of phosphorylated tau in the cortex, hippocampus, and SC in an age-dependent fashion (Götz et al., 2001; Ni et al., 2020). Hemizygous P301L mice were used for breeding to generate homozygous P301L mice. All mice were maintained under specific pathogen-free conditions. Animals were housed in ventilated cages inside a temperature-controlled room (22°C, 50% air humidity), under a 12-h dark/light cycle. Each cage housed up to five mice. Paper tissue and red Tecniplast mouse house^®^ (Tecniplast, Milan, Italy) shelters were placed in cages as environmental enrichments. Pelleted food (3437PXL15, CARGILL) and acidified water were provided *ad libitum*.

### Sample Preparation

All experiments were performed in accordance with the Swiss Federal Act on Animal Protection and were approved by the Cantonal Veterinary Office Zurich (permit number: ZH044/19). All animal experiments are reported in compliance with the ARRIVE 2.0 guidelines. A total of eight male and eight female hemizygous, eight male and seven female homozygous P301L mice and eight male and nine female non-transgenic littermates between 8.5 and 9 months of age were used. Mice were intracardially perfused under deep anesthesia with sodium pentobarbital (100 mg/kg body weight). Mice were perfused intracardially with 10 ml of 0.1 M phosphate buffered saline (PBS) followed by 4% paraformaldehyde in 0.1 M PBS (pH 7.4). Whole vertebral columns were dissected and post-fixed in 4% paraformaldehyde in 0.1 M PBS (pH 7.4) for 6 days and stored in 0.1 M PBS at 4°C thereafter until usage. Samples were placed in a 20 ml syringes filled with perfluoropolyether (Fomblin Y, LVAC 16/6, average molecular weight 2,700, Sigma-Aldrich, USA).

### Magnetic Resonance Imaging Protocol

Data were acquired on a 7/16 small animal MR Pharmascan (Bruker BioSpin AG, Ettlingen, Germany) equipped with an actively shielded gradient set of 760 mT/m and 80 μs rise time, operated by a Paravision 6.0 software platform (Bruker BioSpin). A transmit/receive quadrature birdcage coil with an inner diameter of 23 mm (Bruker BioSpin AG) was used.

First, a T_2_-weighted Turbo Spin Echo sequence was acquired with 10 slices, filed-of-view of 20 mm × 10 mm, matrix dimension 256 × 128, with a nominal voxel resolution of 78 μm × 78 μm was acquired for anatomical orientation and geometrical planning. Imaging parameters were: repetition time of 1,500 ms, an echo time of 6 ms, 2 averages, and a RARE factor of 4 in an acquisition time of 1 min 12 s. Then, T_1_-weighted structural images were acquired with a 3D fast low angle shot (FLASH) sequence with a field of view of 20 mm × 20 mm × 10 mm, matrix dimension of 400 × 400 × 200, resulting in a nominal voxel resolution of 50 μm × 50 μm × 50 μm. In some cases, two field-of-views were acquired to cover the area of interest (C1-C6). The following imaging parameters were chosen: a repetition time of 50 ms, an echo time of 10 ms, 10 averages, within a total scan time of 12 h and 12 min (1 field-of-view) or 24 h and 24 min (2 field-of-views). A field map was acquired with a field-of-view of 15 mm × 15 mm × 15 mm, matrix dimensions of 64 × 64 × 64 to give a resolution of 234 μm × 234 × 234 μm, repetition time 20 ms, echo time 2 ms, 8 averages, within an acquisition time of 10 min 55 s, for using the MAPSHIM protocol for shimming. Field-of-view suppression was used to eliminate signal from regions outside the field-of-view.

### Image Analysis

**Figure 1:**
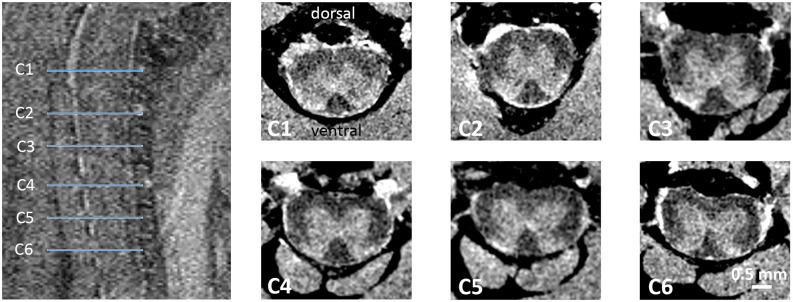
*Ex vivo* MRI of the mouse cervical SC. T_2_-weighted sagittal spin echo images of the upper cervical SC were used for anatomical planning. On axial T_1_-weighted images of the 3D dataset taken at the location C1–C6 (blue lines) were assessed for morphometric analysis.

MR images were analyzed in a validated, open-source, FDA-approved medical imaging program (Osirix MD, v12.0, Geneva, Switzerland; Rosset et al., 2004) by a person blinded to the genotype and gender. Upon inspection of sagittal and coronal image reformations and by using an anatomical atlas (Watson et al., 2009) for guidance, representative axial T_1_-weighted image slices matching C1–C6 SC segments were identified (Figure 1). For each SC segment, three to four consecutive and representative axial image slices were considered for further analysis. Specifically, using freehand region-of-interest (ROI) measurements, the total cross-sectional area, and the gray and white matter anterior-posterior width and left-right width were measured for a given axial slice by manually outlining relevant anatomical structures. The values were subsequently averaged across all three (or four) axial slices, thus rendering a single score per measurement category and SC segment for a given mouse.

### Immunohistochemistry

For immunohistochemistry, three female hemizygous, three female homozygous P301L mice and three female non-transgenic littermates were analyzed. Fixed tissues were cryoprotected in 30% w/v sucrose dissolved in a 0.1 M sodium phosphate buffer for 24–72 h at 4°C. Following cryoprotection, SCs were dissected and the cervical enlargement of each was embedded in Neg-50 freezing medium at −20°C. SCs were embedded upright to allow transverse sections to be taken. Embedded SCs were mounted on a freezing microtome (Hyrax KS-34) and were sectioned at 60 μm with a sliding blade (Zeiss S-30). SC sections were either stored in antifreeze solution, containing 50 mM sodium phosphate buffer, 30% ethylene glycol, 15% glucose, and sodium azide (200 mg/L), or processed directly for immunostaining.

Sections were rinsed three times in 0.1 M PB for 10 min, followed by a 30 min incubation in 50% ethanol. Sections were then rinsed three times for 10 min each with PBS containing extra salt (8 g NaCl per liter PBS), before being incubated for 2 days at 4°C in primary antibodies against NeuN and ionized calcium-binding adapter molecule 1 (Iba-1) to label neurons and resident microglia respectively (primary antibodies suspended in PBS extra salt, 10% v/v normal donkey serum, 0.3% v/v Triton-X). The sections were then rinsed three times for 10 min each in PBS extra salt before being incubated overnight at 4°C in species specific secondary antibodies (suspended in PBS extra salt with 0.3% v/v Triton-X). The following day the tissues were rinsed three times in PBS extra salt, counterstained with DAPI, and mounted on microscope slides in DAKO antifade mounting medium. A list of antibodies used in this study can be found in Table 1.

**Table 1:** List of primary and secondary antibodies used in this study.

**Antibody**	**Host**	**Supplier/Source**	**Cat#/RRID**	**Dilution**
NeuN	Guinea pig	Synaptic systems	266004/AB_2619988	1:1,000
Iba-1	Rabbit	Wako	019-19741/AB_839504	1:2,000
Guinea pig-A647	Donkey	Jackson ImmunoResearch Laboratories	706-469-148/-	1:500
Rabbit	Donkey	Jackson ImmunoResearch Laboratories	711-546-152/AB_2340619	1:500

### Confocal Image Acquisition and Image Analysis

All images were acquired on a Zeiss lsm 800 microscope with Zen blue software. Image stacks were taken with 5 μm z-spacing through a 20× oil immersion objective lens up to a depth of 20 μm from the surface of the section. For analysis, a tile scan was taken to encompass all white and gray matter from the dorsal and ventral horns for half of the SC (hemisection). For all experiments, identical scan settings were used, such as laser power, digital gain, and image resolution. Orthogonal z-projections were produced for each image (four confocal scans at 5 μm z-spacing), which were then analyzed using ImageJ.

Image projections were imported into ImageJ and the gray matter and white matter were delineated using the area or NeuN and dense DAPI staining to identify the gray matter. Mean pixel intensities were measured for the gray and white matter for NeuN and Iba-1 staining, and the size of each of these areas was determined. Between three and four sections were analyzed for each animal (*n* = 3 per group).

### Statistical Analysis

For assessing anatomical variation and effect of gender on morphometric metrics, we performed a two-way ANOVA with segments and gender as independent variables and cross-sectional areas, gray matter area and white matter area as dependent variable. *Post hoc* analysis (Holm-Sidak method) was used to assess interaction between groups. Comparisons of immunohistochemical stainings between group means were made using a one-way ANOVA with Bonferroni *post hoc* correction for multiple comparisons.

## Results

### Morphometric Measurements in the Normal Cervical Cord

**Figure 2:**
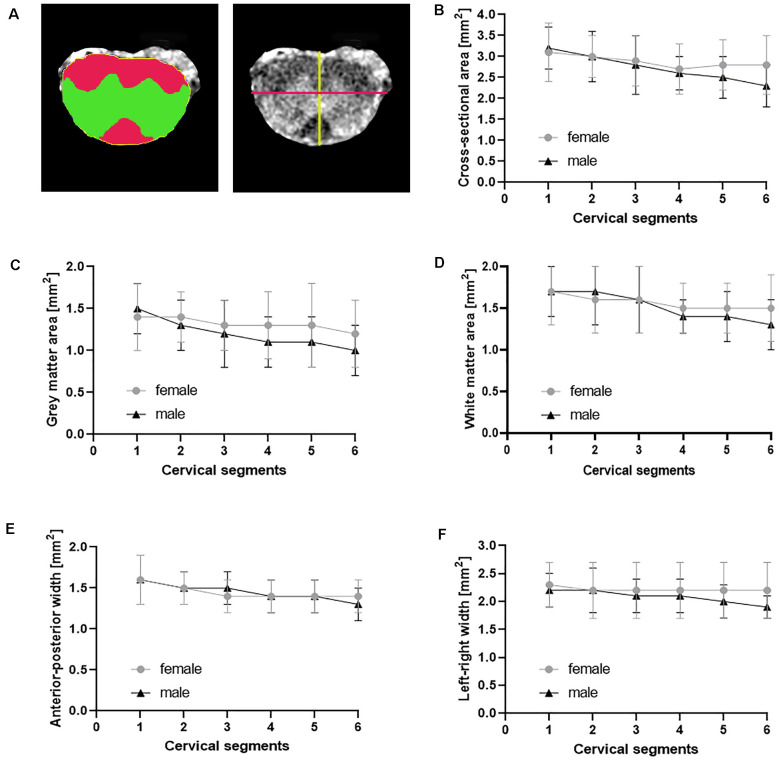
Morphometric measures in normal cervical SC. The cross-sectional area **(A** yellow, **B)**, gray matter area **(A** green, **C)**, white matter area **(A** red, **D)** and the anterior-posterior width **(A** yellow line, **E)** and the left-right width **(A** magenta line, **F)** were measured from axial T_1_-weighted images of female (*n* = 9) and male (*n* = 8) non-transgenic littermates. Shown are mean ± standard deviation for each cervical spinal cord segment.

We first assessed morphometric metrics of the normal cervical SC in non-transgenic littermates (Figure 2, Table 2). Cross-sectional areas decreased from level C1 to C4 as described (Sengul and Watson, 2012). Similarly, gray matter and white matter areas decreased from segment C1 to C4. Values were starting to increase from C5 to C6, encompassing the cervical enlargement. Anterior-posterior and left-and right widths were decreasing from segment C1 to C6.

**Table 2:** Morphometric measures.

**Total cross-sectional area [mm^2^]**		**C1**	**C2**	**C3**	**C4**	**C5**	**C6**
**NTL**	**Female**	3.1 ± 0.7	3.0 ± 0.5	2.9 ± 0.6	2.7 ± 0.6	2.8 ± 0.6	2.8 ± 0.7
	**Male**	3.2 ± 0.5	3.0 ± 0.6	2.8 ± 0.7	2.6 ± 0.4	2.5 ± 0.5	2.3 ± 0.5
**Hemizygote**	**Female**	3.2 ± 0.5	2.9 ± 0.7	2.8 ± 0.7	2.6 ± 0.5	2.6 ± 0.7	2.5 ± 0.7
	**Male**	3.4 ± 0.7	3.1 ± 0.6	2.9 ± 0.6	2.8 ± 0.6	2.7 ± 0.5	2.7 ± 0.6
**Homozygote**	**Female**	2.8 ± 0.8	2.5 ± 0.8	2.3 ± 0.9	2.2 ± 0.9	2.1 ± 1.0	1.9 ± 0.9
	**Male**	2.9 ± 0.5	2.8 ± 0.5	2.7 ± 0.6	2.5 ± 0.6	2.5 ± 0.6	2.5 ± 0.5
**Gray matter area [mm^2^]**						
		**C1**	**C2**	**C3**	**C4**	**C5**	**C6**
**NTL**	**Female**	1.4 ± 0.4	1.4 ± 0.3	1.3 ± 0.3	1.3 ± 0.4	1.3 ± 0.5	1.2 ± 0.4
	**Male**	1.5 ± 0.3	1.3 ± 0.3	1.2 ± 0.4	1.1 ± 0.3	1.1 ± 0.3	1.0 ± 0.3
**Hemizygote**	**Female**	1.5 ± 0.3	1.3 ± 0.3	1.2 ± 0.3	1.1 ± 0.3	1.1 ± 0.3	1.1 ± 0.4
	**Male**	1.7 ± 0.5	1.5 ± 0.3	1.3 ± 0.3	1.3 ± 0.4	1.2 ± 0.3	1.2 ± 0.4
**Homozygote**	**Female**	1.2 ± 0.4	1.0 ± 0.4	0.9 ± 0.4	1.0 ± 0.6	0.8 ± 0.5	0.8 ± 0.6
	**Male**	1.4 ± 0.4	1.3 ± 0.4	1.2 ± 0.4	1.3 ± 0.4	1.2 ± 0.4	1.2 ± 0.4
**White matter area [mm^2^]**						
		**C1**	**C2**	**C3**	**C4**	**C5**	**C6**
**NTL**	**Female**	1.7 ± 0.4	1.6 ± 0.4	1.6 ± 0.4	1.5 ± 0.3	1.5 ± 0.3	1.5 ± 0.4
	**Male**	1.7 ± 0.3	1.7 ± 0.4	1.6 ± 0.4	1.4 ± 0.2	1.4 ± 0.3	1.3 ± 0.3
**Hemizygote**	**Female**	1.8 ± 0.3	1.6 ± 0.4	1.6 ± 0.4	1.5 ± 0.3	1.5 ± 0.4	1.4 ± 0.5
	**Male**	1.7 ± 0.4	1.6 ± 0.4	1.5 ± 0.4	1.5 ± 0.4	1.5 ± 0.3	1.6 ± 0.4
**Homozygote**	**Female**	1.6 ± 0.4	1.5 ± 0.4	1.4 ± 0.5	1.3 ± 0.4	1.3 ± 0.5	1.1 ± 0.4
	**Male**	1.6 ± 0.1	1.5 ± 0.3	1.4 ± 0.2	1.2 ± 0.3	1.3 ± 0.2	1.3 ± 0.2
**Anterior-posterior width [mm]**						
		**C1**	**C2**	**C3**	**C4**	**C5**	**C6**
**NTL**	**Female**	1.6 ± 0.3	1.5 ± 0.2	1.4 ± 0.2	1.4 ± 0.2	1.4 ± 0.2	1.5 ± 0.2
	**Male**	1.6 ± 0.3	1.5 ± 0.2	1.5 ± 0.2	1.4 ± 0.2	1.4 ± 0.2	1.3 ± 0.3
**Hemizygote**	**Female**	1.6 ± 0.3	1.5 ± 0.2	1.4 ± 0.2	1.4 ± 0.2	1.4 ± 0.2	1.4 ± 0.2
	**Male**	1.6 ± 0.4	1.5 ± 0.2	1.5 ± 0.2	1.5 ± 0.2	1.4 ± 0.2	1.4 ± 0.2
**Homozygote**	**Female**	1.4 ± 0.2	1.3 ± 0.3	1.3 ± 0.3	1.2 ± 0.3	1.2 ± 0.3	1.2 ± 0.3
	**Male**	1.4 ± 0.2	1.4 ± 0.2	1.4 ± 0.2	1.3 ± 0.2	1.3 ± 0.2	1.3 ± 0.2
**Left-right width [mm]**							
		**C1**	**C2**	**C3**	**C4**	**C5**	**C6**
**NTL**	**Female**	2.3 ± 0.4	2.2 ± 0.5	2.2 ± 0.5	2.2 ± 0.5	2.2 ± 0.5	2.2 ± 0.5
	**Male**	2.2 ± 0.3	2.2 ± 0.4	2.1 ± 0.3	2.1 ± 0.3	2.0 ± 0.3	1.9 ± 0.2
**Hemizygote**	**Female**	2.5 ± 0.2	2.4 ± 0.3	2.3 ± 0.4	2.2 ± 0.3	2.3 ± 0.3	2.2 ± 0.4
	**Male**	2.4 ± 0.3	2.3 ± 0.3	2.3 ± 0.2	2.3 ± 0.3	2.2 ± 0.3	2.3 ± 0.3
**Homozygote**	**Female**	2.1 ± 0.4	2.0 ± 0.4	2.0 ± 0.5	1.9 ± 0.5	1.9 ± 0.5	1.8 ± 0.5
	**Male**	2.2 ± 0.2	2.1 ± 0.2	2.1 ± 0.2	2.1 ± 0.3	2.2 ± 0.3	2.1 ± 0.3

*NTL, non-transgenic littermates*.

To test for effects of gender on the morphometric measures, we performed a two-way ANOVA with segment and gender as independent variables and morphometric parameters as a dependent variable. We found no statistically significant two-way interaction between gender and segments for the cross-sectional area, *F*_(5)_ = 0.748, *p* = 0.589. Subsequent analysis of main effects revealed no difference in the cross-sectional area between female and male non-transgenic littermates, *F*_(1)_ = 1.917, *p* = 0.170. A significant difference between cervical segments was found, *F*_(5)_ = 2.790, *p* = 0.022. *Post hoc* analysis showed only differences between C1 and C6, with a difference of the mean = 0.620, *t* = 3.085, *p* = 0.040. Furthermore, there was no statistically significant two-way interaction between gender and segments for gray matter area, *F*_(5)_ = 0.683, *p* = 0.637. Also there were no main effects of gender, *F*_(1)_ = 3.086, *p* = 0.082 or segment, *F*_(5)_ = 2.251, *p* = 0.056 on gray matter area. Similarly, there was no statistically significant two-way interaction between gender and segments for white matter area, *F*_(5)_ = 0.520, *p* = 0.761. No main effects of gender, *F*_(1)_ = 0.347, *p* = 0.558 or segments, *F*_(5)_ = 1.895, *p* = 0.103 was found.

Furthermore, there was no statistically significant two-way interaction between gender and segments for anterior-posterior width *F*_(5)_ = 0.642, *p* = 0.668. And there was no main effects of gender, *F*_(1)_ = 0.101, *p* = 0.752 or segments, *F*_(5)_ = 2.195, *p* = 0.062 on anterior-posterior width. Similarly, there was no statistically significant two-way interaction between gender and segments for left-right width *F*_(5)_ = 0.396, *p* = 0.850. No main effects of gender, *F*_(1)_ = 1.718, *p* = 0.193 or segments, *F*_(5)_ = 0.526, *p* = 0.756 was found. Taken together, we did not find differences in the measured morphometric metrics between individual segments of the cervical SC at level 1–6 and no effect of gender on the measured cord metrics.

### Effect of Tau Pathology on Cervical SC Cross-Sectional Area

We then compared the effect of tau pathology on the morphometric measures (Tables s 2, 3). We compared total cross-sectional area between age-matched hemizygous, homozygous P301L mice, and non-transgenic littermates. There was a statistically significant two-interaction between genotype and gender for total cross-sectional area, *F*_(2)_ = 3.682, *p* = 0.026. Subsequent analysis of the factor genotype between female animals revealed a significant difference between homozygous P301L mice and non-transgenic littermates, with a difference of the mean = 0.577, *t* = 4.263, *p* < 0.001. Cross-sectional areas were reduced across all cervical segments (Table 3). A significant difference between cross-sectional areas was also observed between homozygous and hemizygous P301L mice, with a difference of the mean = 0.473, *t* = 3.402, *p* = 0.002, where values were found reduced (Table 3). No differences were found between hemizygous mice and non-transgenic littermates, with a difference of the mean = 0.104, *t* = 0.797, *p* = 0.426.

**Table 3:** Comparison of morphometric measures.

	Change homozygote to NTL [%]		Change homozygote to hemizygote [%]		Change hemizygote to NTL [%]	
	Female	Male	Female	Male	Female	Male
**Total cross-sectional area**
**C1**	−10.2*	−7.2	−13.6*	−12.0	3.9	5.5
**C2**	−16.5*	−7.2	−15.8*	−8.8	−0.9	1.8
**C3**	−21.0*	−6.1	−19.0*	−7.9	−2.5	1.9
**C4**	−18.5*	−1.3	−13.4*	−10.8	−6.0	10.6
**C5**	−24.0*	1.1	−17.9*	−7.8	−7.4	9.6
**C6**	−31.3*	10.0	−23.7*	−9.2	−9.9	21.1
**Gray matter area**
**C1**	−19.5*	−8.0	−20.6*	−17.8	1.4	11.9
**C2**	−26.9*	−3.9	−23.4*	−12.4	−4.5	9.7
**C3**	−31.8*	0	−27.7*	−8.2	−5.6	8.9
**C4**	−26.1*	13.9	−15.0*	−3.2	−13.1	17.7
**C5**	−37.5*	10.7	−23.4*	−1.5	−18.4	12.4
**C6**	−34.1*	23.5	−22.7*	3.0	−14.7	19.9
**White matter area**
**C1**	−2.2	−6.2	−7.8	−5.8	6.0	−0.3
**C2**	−7.8	−12.3	−9.8	−8.2	2.3	−4.4
**C3**	−11.9	−10.8	−12.1	−7.5	0.2	−3.6
**C4**	−12.0	−13.7	−12.2	−17.8	0.3	4.9
**C5**	−11.6	−6.3	−13.9	−12.8	2.7	7.4
**C6**	−29.0	−0.2	−24.4	−18.2	−6.0	22.1
**Anterior−posterior width**					
**C1**	−12.0	−11.7	−12.0	−12.0	0	0.4
**C2**	−14.9	−7.6	−14.9	−10.1	0	2.8
**C3**	−11.3	−6.7	−11.3	−4.3	0	−2.4
**C4**	−10.6	−3.1	−10.6	−8.8	0	6.3
**C5**	−11.3	−3.0	−11.3	−7.6	0	5.1
**C6**	−21.4	1.3	−15.2	−7.3	−7.3	9.3
**Left−right width**
**C1**	−8.7*	−3.3	−17.1*	−8.7	10.2	6.0
**C2**	−8.8*	−4.5	−14.6*	−7.9	6.8	3.7
**C3**	−8.8*	−1.7	−12.6*	−9.4	4.4	8.4
**C4**	−16.0*	1.4	−15.9*	−7.8	−0.2	10.0
**C5**	−11.8*	6.9	−15.0*	−1.5	3.7	8.5
**C6**	−17.9*	9.9	−15.8*	−6.5	−2.5	17.5

NTL, non-transgenic littermates. *Indicates statistically significant differences between mice of a different genotype and same gender using a two-way ANOVA (*p* < 0.05).

Analysis of the factor genotype between male animals revealed no significant difference between homozygous P301L mice and non-transgenic littermates, with a difference of the mean = 0.067, *t* = 0.499 *p* = 0.618. Similarly, there was no statistically significant difference between homozygous and hemizygous P301L mice, with a difference of the mean = 0.278, *t* = 2.065, *p* = 0.115; and there was no significant difference between hemizygous P301L mice and non-transgenic littermates, with a difference of the mean = 0.210, *t* = 1.567 *p* = 0.223.

### Effect of Tau Pathology on Cervical Gray Matter Area

Statistical analysis of gray matter area between age-matched hemizygous, homozygous P301L mice, and non-transgenic littermates revealed a two-way interaction between genotype and gender, *F*_(2)_ = 8.263, *p* < 0.001 (Tables s 2, 3). Subsequent analysis of the factor genotype between female animals revealed a significant difference between homozygous P301L mice and non-transgenic littermates, with a difference of the mean = 0.387, *t* = 4.934, *p* < 0.001. Cross-sectional areas were decreased in homozygous P301L compared to non-transgenic littermates (Table 3). A significant difference was also observed between homozygous and hemizygous P301L mice, with a difference of the mean = 0.268, *t* = 3.326, *p* = 0.002, with a decrease in values (Table 3). No differences were found between hemizygous mice and non-transgenic littermates, with a difference of the mean = 0.119, *t* = 1.574, *p* = 0.117.

Analysis of the factor genotype between male animals revealed no significant difference between homozygous P301L mice and non-transgenic littermates, with a difference of the mean = 0.0549, *t* = 0.705 *p* = 0.482. In addition, there was no statistically significant difference between homozygous and hemizygous P301L mice, with a difference of the mean = 0.103, *t* = 1.321, *p* = 0.340; and there was no significant difference between hemizygous P301L mice and non-transgenic littermates, with a difference of the mean = 0.158, *t* = 2.025 *p* = 0.126.

### Effect of Tau Pathology on Cervical White Matter Area

Statistical analysis of white matter area between age-matched hemizygous, homozygous P301L mice, and non-transgenic littermates revealed no statistically significant two-way interaction between genotype and gender, *F*_(2)_ = 0.178, *p* = 0.837 (Tables s 2, 3). Subsequent analysis showed no effect of gender, *F*_(1)_ = 0.036, *p* = 0.846. However, analysis demonstrated an effect of genotype, *F*_(2)_ = 7.554, *p* < 0.001. Subsequent analysis of the factor genotype revealed a significant difference between homozygous P301L mice and non-transgenic littermates, with a difference of the mean = 0.159, *t* = 3.051, *p* < 0.005. A significant difference was also observed between homozygous and hemizygous P301L mice, with a difference of the mean = 0.193, *t* = 3.650, *p* < 0.001. No differences were found between hemizygous mice and non-transgenic littermates, with a difference of the mean = 0.034, *t* = 0.661, *p* = 0.509.

### Effect of Tau Pathology on Anterior-Posterior and Left-Right Width

Statistical analysis of the anterior-posterior width between age-matched hemizygous, homozygous P301L mice, and non-transgenic littermates revealed no statistically significant two-way interaction between genotype and gender, *F*_(2)_ = 1.471, *p* = 0.232 (Tables s 2, 3). Subsequent analysis showed no effect of gender, *F*_(1)_ = 2.670, *p* = 0.103. However, analysis demonstrated an effect of genotype, *F*_(2)_ = 11.823, *p* < 0.001. Subsequent analysis of the factor genotype revealed a significant difference between homozygous P301L mice and non-transgenic littermates, with a difference of the mean = 0.136, *t* = 3.998, *p* < 0.001. A significant difference was also observed between homozygous and hemizygous P301L mice, with a difference of the mean = 0.154, *t* = 4.453, *p* < 0.001. No differences were found between hemizygous mice and non-transgenic littermates, with a difference of the mean = 0.0176, *t* = 0.526, *p* = 0.599.

Statistical analysis of left-right width revealed a two-interaction between genotype and gender, *F*_(2)_ = 4.144, *p* < 0.017 (Tables s 2, 3). Subsequent analysis of the factor genotype between female animals revealed a significant difference between homozygous P301L mice and non-transgenic littermates, with a difference of the mean = 0.268, *t* = 3.676, *p* < 0.001. Anterior-posterior widths were decreased in homozygous P301L compared to non-transgenic littermates (Table 3). A significant difference was also observed between homozygous and hemizygous P301L mice, with a difference of the mean = 0.352, *t* = 4.701, *p* < 0.001, with a decrease in values (Table 3). No differences were found between hemizygous mice and non-transgenic littermates, with a difference of the mean = 0.084, *t* = 1.195, *p* = 0.233.

Analysis of the factor genotype between male animals revealed no significant difference between homozygous P301L mice and non-transgenic littermates, with a difference of the mean = 0.0248, *t* = 0.343 *p* = 0.732. In addition, there was no statistically significant difference between homozygous and hemizygous P301L mice, with a difference of the mean = 0.161, *t* = 2.226, *p* = 0.053. There was a significant difference between hemizygous P301L mice and non-transgenic littermates, with a difference of the mean = 0.186, *t* = 2.569 *p* = 0.032.

### Marked Neurodegeneration and Neuroinflammation in the Gray Matter of the Cervical SC

**Figure 3:**
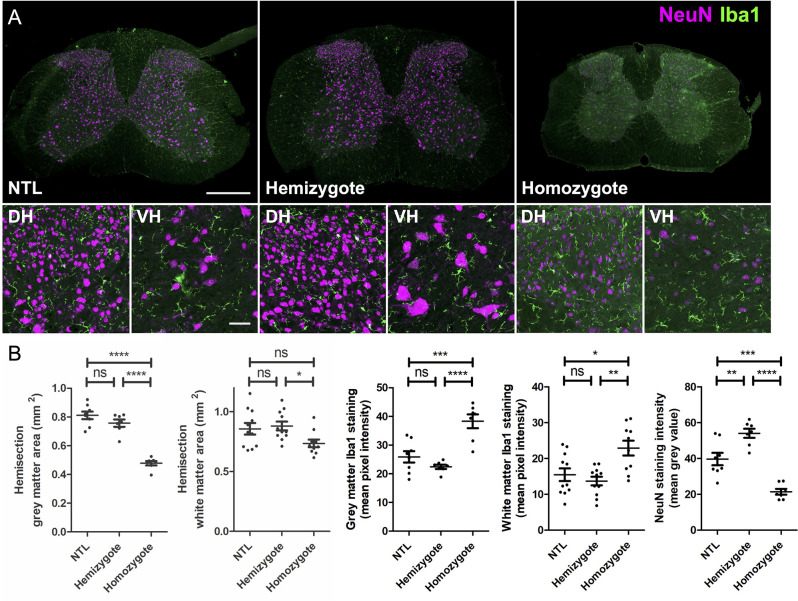
Atrophy and microglia activation in SCs of P301L homozygous mice. **(A)** Representative images of cervical SC sections after immunohistochemistry from non-transgenic littermates (NTL), hemizygous, and P301L homozygous animals. Overview images taken at 20× magnification and enlargements of the dorsal horns (DH) and ventral horns (VH) taken at 40× magnification. All images are a projection of two optical sections at 5 μm z spacing, scale bar = 200 μm for overview images and 50 μm for enlargements. **(B)** Quantification of tissues stained with NeuN and Iba-1. For analysis a tile scan was taken for half of the SC (hemisection). Cervical SCs from P301L homozygous animals had a significantly smaller gray (*p* < 0.0001) and white matter areas (*p* < 0.05), higher Iba-1 staining intensity in the gray (*p* < 0.0001) and white matter (*p* < 0.01), and lower NeuN staining intensity compared to hemizygous animals (One-way ANOVA, with Bonferonni *post hoc* correction). *p < 0.05; **p < 0.01; ***p < 0.001; ****p < 0.0001; ns, not significant.

Immunohistochemical analysis revealed marked differences in NeuN and Iba-1 stainings between NTLs and hemizygous and homozygous P301L mice (Figure 3). Quantification of stainings showed reduced gray matter areas in the cervical SCs from P301L homozygous animals compared to hemizygous P301L mice and NTLs (Figure 3B). Similarly, the expression of the neuronal marker NeuN was reduced in P301L homozygous animals compared to hemizygous P301L mice and NTLs, while hemizygous P301L mice displayed higher NeuN levels than NTL. White matter areas were reduced in homozygous P301L compared to hemizygous littermates. Neuroinflammation was evident by increased Iba-1 staining in homozygous P301L mice compared to hemizygous littermates and NTLs.

## Discussion

We characterized morphological changes of the cervical SC in the P301L mouse model of tauopathy. Computing morphometric metrics from structural high-resolution *ex vivo* MRI data revealed atrophy of the cervical segments C1–6. Importantly, we found that the observed changes depend both on gender and transgene expression levels. Immunohistochemical analysis revealed atrophy in conjunction with neuroinflammation in the cervical SC of P301l mice.

Structural MRI has been used to assess morphology of the normal SC in humans (Sherman et al., 1990; Kato et al., 2012; Engl et al., 2013; Ulbrich et al., 2014; Papinutto et al., 2015), but values for the mouse cervical SC have so far not been reported. Thus, we first determined morphometric metrics of the cervical SC in non-transgenic littermates (Figure 2, Table 2). Cross-sectional area, gray matter and white matter areas as well as anterior-posterior and left-right widths followed the described anatomical structure of the cervical SC (Sengul and Watson, 2012). Human MRI studies have suggested sexual dimorphism of the cervical total cross-sectional, gray and white matter areas in adults, with men having larger values compared to women (Ulbrich et al., 2014; Papinutto et al., 2015). It has been suggested that this difference reflects differences in size between genders as SC measures are highly correlated with total cranial volume and vertebral dimensions (Papinutto et al., 2015). However, other studies have not seen differences in morphometric measures between males and females (Sherman et al., 1990; Kato et al., 2012; Engl et al., 2013). In our study, we did not observe significant differences in any of the analyzed morphometric measures between male and female non-transgenic mice. Male mice of the C57Bl6 background are heavier than females (Kovács and Pearce, 2013), which however was unrelated to higher morphometric measures in cervical cord. However, the small group sizes, compared to clinical MRI SC studies, might have prevented us from detecting a sexual dimorphism in cervical SC measures. More systematic studies assessing morphometric measures in male and female mice in larger cohorts should be conducted.

We further characterized morphological changes of the cervical SC related to tau by comparing morphometric metrics of hemizygous and homozygous P301L mice with age-matched non-transgenic littermates. We found clear evidence for atrophy of the cervical SC in P301L mice (Table 3). Thus, we further determined how gender, transgene expression, and anatomical location are related to the observed changes in cervical SC morphology.

While we have not seen an effect of gender in the normal morphology of the cervical SC, we tested for gender differences in tau-related cervical SC atrophy. Gender effects have been reported in the manifestation and progression of a variety of neurological diseases such as stroke (Cordonnier et al., 2017), Parkinson’s disease (Liu et al., 2017), and SC injury (Stewart et al., 2020). Gender differences have also been reported for tauopathies. In Alzheimer’s disease there is not only a higher prevalence in women than men (Plassman et al., 2007; Carter et al., 2012), but it is also associated with a higher tau cerebrospinal fluid level, cortical tau load, and degree of neurodegeneration in females (Filon et al., 2016; Hohman et al., 2018). Moreover, neuroimaging studies in cognitively normal adults and patients with mild cognitive impairment have shown that women exhibit higher cerebral tau accumulation than men, and a different spread of tau pathology across the brain (Buckley et al., 2019; Shokouhi et al., 2020). Sexual dimorphism of the observed phenotype in animal models of tauopathy is not consistently assessed. Specifically, the results from the small number of studies examining the effect of gender vary considerably. A study using rTg4510 mice showed significantly higher levels of hyperphosphorylated tau in females than males, associated with more severe impairment in spatial learning and memory (Yue et al., 2011). In contrast, a study in P301S showed a stronger cognitive deficit, neuropathology, and inflammatory plasma proteins in male transgenic mice (Sun et al., 2020). We have recently reported no differences in the number of cerebral calcifications between male and female P301L mice as observed with susceptibility weighted imaging and phase imaging (Ni et al., 2020). In the present study, we observed a statistically significant two-interaction between genotype and gender for total cross-sectional area, gray matter area and left-right width where subsequent analysis revealed significant differences in this parameter for female mice only (Table 3). No two-way interaction was found for the white matter area and anterior-posterior width. This clearly implicates gender as an important factor in the observed phenotype of cervical SC atrophy.

We have also studied the effect of transgene-dosing on the measured morphometric measures by including both hemizygous and homozygous P301L mice in the study. In a recent brain diffusion tensor imaging study, we found more pronounced and wide-spread microstructural changes in homozygous compared to hemizygous P301L mice (Massalimova et al., 2020). In this study, we observed stronger reduction in total cross-sectional area, and gray and white matter areas in homozygous than in hemizygous P301L mice (Table 3). For anterior-posterior and left-right width there was no obvious differences in the measured reductions between hemizygote and homozygote mice compared to non-transgenic littermates (Table 3). These observations are in line with immunohistochemical analysis, where we have seen lower gray and white matter as well as lower NeuN staining intensities in homozygous P301L mice compared to their hemizygous littermates and NTLs, while there was no difference between these measures in hemizygous P301L mice compared to NTL (Figure 3). Taken together the data shows that high transgene overexpression is a second important contributor to the observed phenotype.

Another aspect constitutes the location of neuropathology in the SC. A neuropathological study in Alzheimer’s disease patients suggested a gradient in the SC tau pathology, with more pronounced tau deposition in the cervical SC, which decreased progressively from the thoracic to the lumbar level (Dugger et al., 2013). In the present study, we have only acquired data from the cervical SC segments C1 to C6 as this area was covered by the birdcage coil. Thus, we have no information about SC morphometric measures for the lowest cervical segments (C7 and C8) as well as the more distal parts of the SC. In the cervical region (C1-C6) we have seen decreases in total cross-sectional area, and gray and white matter area across all segments (Table 3). This is line with a recent study in Alzheimer’s disease patients, which found reduced cross-sectional areas in segments C1 to C5 (Lorenzi et al., 2020). Thus, further studies should aim to elucidate the relationship between morphometric measures in the SC and tau pathology in other SC regions and assess how cervical SC atrophy progresses during the disease course. Moreover, structural MRI studies have shown regional atrophy in the brain of patients with tauopathies (Lieberman et al., 1998; Price et al., 2004; Ghetti et al., 2008; Holland et al., 2009; Shi et al., 2009; Tokumaru et al., 2009; Rohrer et al., 2010; Tabatabaei-Jafari et al., 2019; Lorenzi et al., 2020). Cerebral atrophy was also seen in preclinical MRI studies of mouse models of tauopathy (Xie et al., 2010; Yue et al., 2011; Holmes et al., 2017; Ma et al., 2019). In a recent study, we have not seen cerebral atrophy in hemizygous P301L mice at 10–12 and 19–21 months of age (Kindler et al., 2021), but we have not yet studied cerebral morphology in homozygous P301L mice. As the observation of cervical SC atrophy is a novel finding, the relationship between cerebral and cervical SC atrophy in models of tauopathy needs to be further investigated.

Neuropathological studies in the SC have shown that the tau pathology affects both gray and white matter (Ishihara et al., 1999; Spittaels et al., 1999; Probst et al., 2000; Verkkoniemi et al., 2001; Allen et al., 2002; Higuchi et al., 2002; Zehr et al., 2004; Lin et al., 2005; Rudzinski et al., 2008; Dugger et al., 2013). While we have seen also reductions in the white matter area in homozygous and hemizygous P301L mice compared to non-transgenic littermates, reductions were stronger in the gray matter (Table 3). This is in agreement with immunohistochemical analysis where we have seen significant reductions in the white matter area in homozygous P301L mice compared to hemizygous P301L mice and NTLs, but the reductions in gray matter area were more pronounced (Figure 3). This suggests that the overall observed atrophy in the cervical SC is largely due to degeneration of the gray matter. We have also observed that the degenerative changes were accompanied by inflammation, as shown by increased Iba-1 expression in the gray and white matter in homozygous P301L mice (Figure 3), confirming previous reports in different mouse tauopathy mouse models (Allen et al., 2002; Higuchi et al., 2002; Lin et al., 2005; Yoshiyama et al., 2007; Rudzinski et al., 2008).

In this study, we acquired structural T_1_-weighted data of the cervical SC *ex vivo*. *Ex vivo* MRI offers distinct advantages over *in vivo* MRI studies (Stroman et al., 2014). First, the small size of mouse SC requires a high spatial resolution of the SC to reliably depict anatomical details. *Ex vivo* MRI allowed long data acquisition, which we used to achieve a 50 μm isotropic resolution with high signal-to-noise ratio. Second, motion of the SC and its surrounding tissue by breathing and cerebrospinal fluid flow can lead to motion artifacts during *in vivo* MRI acquisition, which does not occur in *ex vivo* acquisitions. Third, inhomogeneities in magnetic susceptibility from air-tissue interfaces, result in image distortions and a loss of signal intensity. Embedding samples in perfluoropolyether effectively eliminates air from around the sample. In similar preparations of the head, we have recently shown microstructural changes in the gray and white matter of the brain in P301L mice of the same age as used in the current study with diffusion tensor imaging (Massalimova et al., 2020) and cerebral calcifications with susceptibility weighted imaging and phase imaging (Ni et al., 2020). Future studies should aim to assess neurodegenerative changes in the cervical SC with MRI *in vivo*. Furthermore, advanced quantitative MRI protocols have been applied to the SC and have the potential to provide sensitive and specific markers of SC pathology (Seif et al., 2018). Such tools may help to improve the diagnosis, stratification, and monitoring of patients with tauopathies.

In summary, we provide evidence for cervical SC atrophy in P301L mice, that depends both on gender and transgene expression levels. Future MRI studies in mouse models of tauopathies should focus on the role of cervical SC in the disease progression and symptology.

## Data Availability Statement

The raw data supporting the conclusions of this article will be made available by the authors, without undue reservation.

## Ethics Statement

The animal study was reviewed and approved by Cantonal Veterinary Office Zurich. Written informed consent was obtained from the owners for the participation of their animals in this study.

## Author Contributions

JK conceived and designed the study and wrote the manuscript. TS and RG performed the experiments. TS, RG, and JK analyzed the data. TS, RG, RN, PF, HZ, and JK interpreted the results. All authors contributed to the article and approved the submitted version.

## Conflict of Interest

The authors declare that the research was conducted in the absence of any commercial or financial relationships that could be construed as a potential conflict of interest.

## Publisher’s Note

All claims expressed in this article are solely those of the authors and do not necessarily represent those of their affiliated organizations, or those of the publisher, the editors and the reviewers. Any product that may be evaluated in this article, or claim that may be made by its manufacturer, is not guaranteed or endorsed by the publisher.
